# Falsely prolonged activated partial thromboplastin time – a pre- and post-analytical issue

**DOI:** 10.11613/BM.2019.011001

**Published:** 2018-12-15

**Authors:** Charlotte Gils, Pernille Just Vinholt, Mads Nybo

**Affiliations:** Department of Clinical Biochemistry and Pharmacology, Odense University Hospital, Odense, Denmark

**Keywords:** activated partial thromboplastin time, haemolysis, lipemia, post-analytical errors, pre-analytical errors

## Abstract

This case highlights two common pre-analytical problems identified in routine coagulation testing of activated partial thromboplastin time (aPTT), which were overlooked because of a concurrent flag code indicating no coagulation and the result was replaced by asterisks. It concerns a boy with gastrointestinal bleeding and prolonged aPTT > 300 seconds, which raised the suspicion of haemophilia. When all other coagulation parameters (including specific coagulation factors VIII and IX) turned out to be normal, aPTT was re-measured using another analysis principle, which revealed a normal aPTT. The primary aPTT result turned out to be aborted due to concurrent haemolysis and lipaemia, but was erroneously interpreted as prolonged coagulation. The lesson is awareness of the possibility of numerous flag codes on the same sample overruling each other, and awareness on the responsibility in the post-analytical phase that must be carried by increased educational focus and by the manufacturers.

## Introduction

A laboratory test result can be affected by interfering factors throughout the entire test process, which can be divided into the pre-analytical, analytical and post-analytical phase; all these phases must therefore be taken into account in order to interpret a test result correctly (*1*). A lot of effort is put into minimizing medical errors arising from laboratory diagnostics, especially errors emerging in relation to the pre-analytical phase as they constitute the vast majority with a reported frequency of 70% of total errors in laboratory testing (*2*). Among these, endogenous interference is a frequent reason for inappropriate quality of the specimen. Also, the post-analytical phase related to reporting test results and interpretation of these can be source of errors in laboratory testing, although less data are available on this area.

Among pre-analytical errors, interference is frequently observed from haemoglobin, bilirubin and lipaemia (HIL), the so-called HIL indices. Importantly, the complexity of haemostasis and intricacy of analytical methods makes coagulation testing more vulnerable to HIL interferences than other areas of *in vitro* laboratory diagnostics (*1*, *3*). Activated partial thromboplastin time (aPTT) estimates the activity of the intrinsic and common pathways of coagulation by measuring the time in seconds required for a fibrin clot to form in a plasma sample with appropriate amounts of calcium chloride and a thromboplastin reagent. The analysis is mostly performed as a part of series of screening tests for bleeding tendency (*4*). In a routine setting, aPTT can be measured electromechanically or with an optical technique (*5*). The electromechanical measurement principle is unaffected by spectral interferences from haemolysis, but biological interference due to haemolysis is still tangible and cannot be overcome (*6*). The use of an optical technique and multiple wavelengths offers the possibility of a simultaneous check for endogenous interfering substances such as haemoglobin, bilirubin or lipaemia – but the test itself is susceptible to those interferences (*5*). The biological interference from haemoglobin may be just as important as the analytical interference in coagulation testing because it affects both optical and mechanical instrumentation and is often overlooked (*1*). We here present an example of interference from lipaemia and haemolysis on an aPTT measurement, where the handling of this information critically influenced the report of the test result in the post-analytical phase.

## Case report

A young boy was admitted with gastrointestinal bleeding. As part of the diagnostic workup different coagulation parameters were measured and revealed normal international normalized ratio (INR) of 1.10 (reference range < 1.2) and normal fibrinogen activity of 1.87 g/L (ref. 1.77 – 4.29 g/L) but a prolonged aPTT > 300 seconds (ref. 22 - 28 seconds). Due to a hereof relevant suspicion of haemophilia (and an expected high bleeding risk based on the aPTT result), coagulation factors (F) VIII and IX were measured and were normal: FVIII was 0.92 x 10^3^ IU/L (ref. 0.60 - 1.50) and FIX 1.89 x 10^3^ IU/L (ref. 0.60 - 1.50). Coagulation parameters were all measured on Sysmex CS-5100 (Siemens Healthcare A/S, Ballerup, Denmark) using optical technology.

To unravel this discrepancy, aPTT was measured on an alternative coagulation instrument, STA-R (Triolab AS, Broendby, Denmark), using an electromechanical measurement principle, which revealed a normal aPTT of 34 seconds (ref. 27 - 40 seconds).

Due to normal INR, normal fibrinogen, normal FVIII and FIX and a normal aPTT measured with electromechanical technique the troubleshooting was focused on the aPTT result > 300 seconds using the optical technique. Based on the normal aPTT result from the same specimen with another method, interference from heparin was excluded. Factors VIII, IX and aPTT measured with a mechanical method were analysed on the same specimen as the previous analyses and not a redrawn sample.

**Figure 1:**
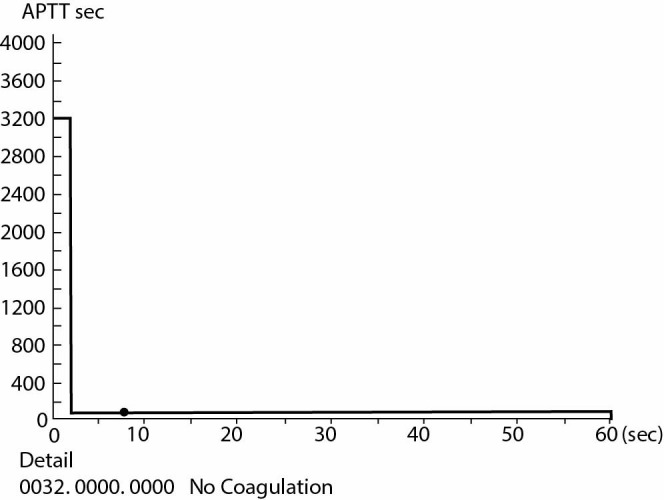
The aPTT measurement curve in the patient sample. X-axis: time (s), Y-axis: aPTT-value (s). aPTT - activated partial thromboplastin time.

**Figure 2:**
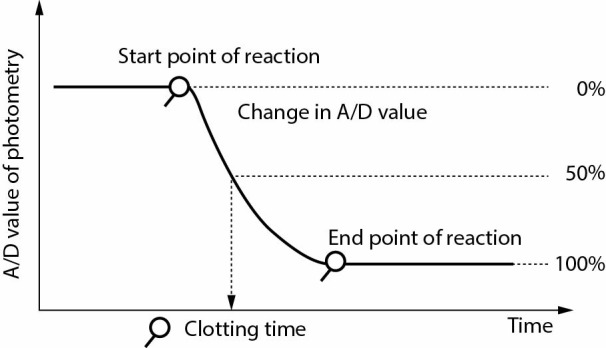
A normal aPTT measurement curve. aPTT - activated partial thromboplastin time.

The Graph Detail Print from the patient sample revealed a clotting curve as shown in Figure 1. The shape of the curve indicates that no clotting point is detectable. For comparison, a normal curve is shown in Figure 2. Due to the abnormal curve, the flag code *“no coagulation”* was added automatically from the analysis equipment and the result of the aPTT replaced by asterisks (*****). In such a situation, the local standard operating procedure (SOP) describes that the laboratory technician must check if the sample is coagulated, and only if the sample is *not* coagulated, an aPTT result > 300 seconds can be released. In this case, the sample was not coagulated and the situation was actually handled according to the SOP. Unfortunately, the local SOP did not describe action steps in case of the error *“no coagulation”* precisely enough. According to the manufacturer’s check algorithm, the sample must be checked for spurious etiology (*i.e*. pre-analytical errors or unknown anticoagulant therapy) in case of a *“no coagulation”* message. If HIL notifications appear and the result of aPTT is replaced by asterisks, the test result must however *not* be released, but instead replaced by the relevant interference code. In this case, the Graph Detail Print also revealed two other flag codes, namely *“haemolysed sample”* and *“lipemic sample”*. Consequently, the *“no coagulation”* code should have been overruled by the interference codes for haemolysis and lipaemia, which unfortunately passed unnoticed. This was possible because the possibility of a flag code *“no coagulation”* in the presence of increased HIL indices was not anticipated, and the combination of these was therefore not comprised by the SOP.

## What happened?

The aPTT result was corrected to 34 seconds in the medical report and the clinicians were informed on the laboratory error. Also, the clinicians were informed on the highly lipaemic and haemolysed sample. Plasma triglycerides measurement was consequently performed revealing severely increased triglycerides of 7.59 mmol/L (ref. < 2.00 mmol/L).

Also, the local SOP was corrected: In case of other codes than “*no coagulation”* and a result replaced by asterisks, the aPTT result must *never* be released.

## Discussion

Inappropriate handling of test results, either by the laboratory or by the clinician doing the interpretation, can have harmful consequences (*7*). Especially, errors related to coagulation testing can have clinically significant consequences if placing the patient at risk of bleeding or thrombosis (*7*). An aPTT > 300 seconds is associated with increased bleeding risk unless the reason is presence of lupus anticoagulant (LA), which is known to cause false prolongation of aPTT (*8*). In our case, the aPTT prolongation erroneously indicated that the patient had an increased bleeding risk, which potentially could have influenced the clinical decision-making and postponed further investigations of the patient’s symptoms, if these had included operative procedures contraindicated in case of increased bleeding risk.

Influence of haemolysis and lipaemia on coagulation testing represents well-known diagnostic challenges. The complexity of haemostasis and also of the analytical methods makes the coagulation analyses vulnerable to these interferences (*1*). For this reason, it is also explicitly stated in standard guideline H21-A5 from the Clinical and Laboratory Standards Institute (CLSI) that haemolysed, icteric or lipaemic samples represent a serious challenge in plasma-based coagulation and molecular haemostasis assays (*3*).

The degree of interference from HIL varies depending on the aPTT method used (*9*). Evidence of influence from haemolysis on routine coagulation testing shows that aPTT can be prolonged in haemolysed patient samples but shortened in samples from healthy volunteers subjected to mechanical haemolysis when using a MDA-II analyser (*10*). Shortening of aPTT was also observed in spiked samples from healthy volunteers using Behring Coagulation System (Dade-Behring, Marburg, Germany), whereas spiking patient samples with normal aPTT with haemolytic haemoglobin prolonged the aPTT value using Sysmex CS-2100i (*9*, *11*).

Regarding lipaemia, research groups have compared routine coagulation testing on optical instruments with those of a mechanical clot detection-based analyser. Comparison of aPTT measurements from Sysmex CA-6000 and STA-R in lipaemic samples (triglycerides concentration ranging between 2.42 and 18.70 mmol/L) found a high correlation between aPTT results from the two analysers (r = 0.988) (*12*). In a large volume hospital laboratory, Sysmex CA-1500 compared to STA-R also showed a high correlation between aPTT in turbid samples (*13*). Thus, current evidence does not indicate any significant impact of turbidity on photo-optical detection as well as mechanical detection on aPTT measurements, whereas the literature regarding haemolysis and aPTT results is more inconsistent.

When handling common interferences related to sample integrity such as HIL, clinical laboratories often rely on the documentation of HIL estimates and interference levels given by the manufacturer. However, it is important for the laboratories to evaluate this information for clinical practice and patient safety to prevent reporting of erroneous values. Also, the laboratories must consider a way to avoid rejecting too many samples, where the result perhaps could be valuable to the patient despite an increased uncertainty due to *e.g.* haemolysis (*14*). In general, laboratories cannot rely solely on guidelines or manufacturer information to assure correct interpretation – they are obliged to confirm this as part of their mandatory assay verification due to the ISO15189 standard.

Modern instrumentation and improved test performance decreases the risk of analytical errors in laboratory testing, but increasing lab automation is however a challenge when it comes to assurance of sample integrity. Errors observed in reported test results are therefore far more likely to be related to pre- or post-analytical issues, and focus on post-analytical handling of pre-analytical issues are therefore more relevant than ever, especially when implementing new equipment (which was the case here) – and in this regard involvement of the manufacturers are mandatory (*6*). Furthermore, the medical doctors in clinical biochemistry and specialists in laboratory medicine are pivotal in evaluating the influence of pre-analytical issues on the post-analytical phase as they constitute an important academic link between the laboratory and the clinicians. So an optimal handling of pre- as well as post-analytical issues and how to handle or avoid these warrants close cooperation between the manufacturer, the laboratory specialists, and the clinicians.

## What YOU should / can do in your laboratory to prevent such errors

Laboratories must check HIL indices before anything else is done on the sample.
Laboratories must be aware of the possibility of more than one flag code in routine testing, here shown for coagulation tests on Sysmex CS-5100.
If the HIL indices constitute one or more flag codes, the action of these should always overrule the action of any other flag codes if the result is replaced by asterisks when using Sysmex CS-5100.
Flag codes should prompt further investigation of the cause, but the final interpretation and decision must be carried out by the laboratory specialist.
In the increasingly automated laboratory, awareness on pre- and post-analytical issues, especially if they combine, must be strengthened.
